# Resistance to Tyrosine Kinase Inhibitors in Chronic Myeloid Leukemia—From Molecular Mechanisms to Clinical Relevance

**DOI:** 10.3390/cancers13194820

**Published:** 2021-09-26

**Authors:** Raquel Alves, Ana Cristina Gonçalves, Sergio Rutella, António M. Almeida, Javier De Las Rivas, Ioannis P. Trougakos, Ana Bela Sarmento Ribeiro

**Affiliations:** 1 Laboratory of Oncobiology and Hematology, University Clinic of Hematology, Faculty of Medicine (FMUC), University of Coimbra, 3000-548 Coimbra, Portugal; raquel.alves@fmed.uc.pt (R.A.); acgoncalves@fmed.uc.pt (A.C.G.); 2 Group of Environment Genetics and Oncobiology (CIMAGO), Institute for Clinical and Biomedical Research (iCBR), Faculty of Medicine, University of Coimbra, 3000-548 Coimbra, Portugal; 3 Center for Innovative Biomedicine and Biotechnology (CIBB), 3000-548 Coimbra, Portugal; 4 Clinical Academic Center of Coimbra (CACC), 3000-061 Coimbra, Portugal; 5 John van Geest Cancer Research Centre, Centre for Health, Ageing and Understanding Disease (CHAUD), School of Science and Technology, Nottingham Trent University, Nottingham NG11 8NS, UK; sergio.rutella@ntu.ac.uk; 6 Hospital da Luz Lisboa, 1500-650 Lisbon, Portugal; amalmeida@ucp.pt; 7 Faculdade de Medicina, Universidade Católica Portuguesa, 2635-631 Lisbon, Portugal; 8 Cancer Research Center (CiC-IBMCC, CSIC/USAL/IBSAL, Consejo Superior de Investigaciones Científicas (CSIC), University of Salamanca (USAL) and Instituto de Investigación Biomédica de Salamanca (IBSAL)), 37007 Salamanca, Spain; jrivas@usal.es; 9 Department of Cell Biology and Biophysics, Faculty of Biology, National and Kapodistrian University of Athens, 15784 Athens, Greece; itrougakos@biol.uoa.gr; 10 Department of Life Sciences, European University Cyprus, 2404 Nicosia, Cyprus; 11 Hematology Service, Centro Hospitalar e Universitário de Coimbra (CHUC), 3000-061 Coimbra, Portugal

**Keywords:** CML, TKI resistance, epigenetics, immune system, new targeted therapies, patient adherence, bioinformatics and artificial intelligence

## Abstract

**Simple Summary:**

Chronic myeloid leukemia (CML) is a myeloproliferative neoplasia associated with a molecular alteration, the fusion gene *BCR-ABL1*, that encodes the tyrosine kinase oncoprotein BCR-ABL1. This led to the development of tyrosine kinase inhibitors (TKI), with Imatinib being the first TKI approved. Although the vast majority of CML patients respond to Imatinib, resistance to this targeted therapy contributes to therapeutic failure and relapse. Here we review the molecular mechanisms and other factors (e.g., patient adherence) involved in TKI resistance, the methodologies to access these mechanisms, and the possible therapeutic approaches to circumvent TKI resistance in CML.

**Abstract:**

Resistance to targeted therapies is a complex and multifactorial process that culminates in the selection of a cancer clone with the ability to evade treatment. Chronic myeloid leukemia (CML) was the first malignancy recognized to be associated with a genetic alteration, the t(9;22)(q34;q11). This translocation originates the *BCR-ABL1* fusion gene, encoding the cytoplasmic chimeric BCR-ABL1 protein that displays an abnormally high tyrosine kinase activity. Although the vast majority of patients with CML respond to Imatinib, a tyrosine kinase inhibitor (TKI), resistance might occur either de novo or during treatment. In CML, the TKI resistance mechanisms are usually subdivided into BCR-ABL1-dependent and independent mechanisms. Furthermore, patients’ compliance/adherence to therapy is critical to CML management. Techniques with enhanced sensitivity like NGS and dPCR, the use of artificial intelligence (AI) techniques, and the development of mathematical modeling and computational prediction methods could reveal the underlying mechanisms of drug resistance and facilitate the design of more effective treatment strategies for improving drug efficacy in CML patients. Here we review the molecular mechanisms and other factors involved in resistance to TKIs in CML and the new methodologies to access these mechanisms, and the therapeutic approaches to circumvent TKI resistance.

## 1. Introduction

Chronic myeloid leukemia (CML) is a clonal hematopoietic stem cell (HSC) neoplasia characterized by an increase in myeloid lineage cells at all differentiation stages [1]. This myeloproliferative neoplasm has an incidence of 1–2 cases per 100,000 adults, representing approximately 15% of newly diagnosed cases of leukemia in adults [2]. In 2020, in the United States, it was estimated that about 8450 new CML cases were diagnosed, and about 1080 CML patients died. It should be noted that since the introduction of Imatinib in 2001, the annual mortality in CML has decreased from 10 to 20% to 1 to 2%, and the 5-year relative survival between 2011 and 2017 was 70.6% [2,3].

With the discovery of the Philadelphia (Ph) chromosome in 1960 [4], CML was the first human malignancy to be associated with a consistent chromosomal abnormality [5]. This cytogenetic hallmark has origin in the reciprocal translocation between the long arms of chromosomes 9 and 22, leading to a smaller chromosome 22, called chromosome Philadelphia, that is present in 95% of CML patients [6]. This exchange of genetic material establishes the fusion gene *BCR-ABL1*. This fusion gene emerges from the juxtaposition of the proto-oncogene 1 non-receptor tyrosine kinase, *ABL1* gene, at chromosome 9 with the activator of RhoGEF and GTPase, *BCR* gene, on chromosome 22 [7]. This oncogene encodes the oncoprotein BCR-ABL1, which presents aberrant constitutive tyrosine kinase activity being crucial for HSC transformation and leukemia initiation [8]. This activity provides survival signals to the malignant cells, inducing cell proliferation and resistance to programmed cell death [1].

The development of a small molecule with the ability to block the BCR-ABL1 activity dramatically changed the disease course, and CML gradually became a chronic disease [9,10]. This tyrosine kinase inhibitor (TKI) impairs the interaction of BCR-ABL1 and ATP, blocking cell signals and, consequently, reducing cell proliferation and inducing cell death f CML clones. Called the “magic bullet” by Time magazine in 2001, Imatinib was the first TKI approved by the Food and Drug Administration (FDA) and European Medicines Agency (EMA) to treat resistant/refractory CML patients [11] and for newly diagnosed patients just two years later [5].

Currently, five TKIs are approved for CML treatment. These TKIs are Imatinib, Dasatinib, Nilotinib, Bosutinib, and Ponatinib. Imatinib, Dasatinib, Nilotinib, and Bosutinib are the current first-line treatments approved by the FDA and EMA for the treatment of CML [2]. The evolution of these drugs to treat CML, over the last two decades, has been quite remarkable in a continuous fight against resistance. Radotinib is a second generation TKI currently only approved by Korean authorities and Flumatinib was approved at the end of 2019 in China for CML treatment. Nonetheless, in spite of the massive improvement in CML treatment over the last years with the introduction of TKIs, some patients (20–30%) display intrinsic or acquired resistance to treatment during the disease course [4,12].

Resistance to target therapy is a complex and multifactorial process that culminates in the selection of a cancer clone with the ability to evade treatment [13]. In CML, TKIs resistance mechanisms are usually subdivided into BCR-ABL1 dependent and independent mechanisms [14]. However, in therapeutic guidelines, only BCR-ABL1 related mechanisms are taken into consideration for dose adjustments or TKI switch [15]. The persistence of leukemic stem cells (LSCs) and LSC-like phenotype based on BCR-ABL1 protein suppression have also been reported as a main TKI resistance mechanisms [1]. Furthermore, patient adherence to therapy and the compliance with professional instructions are critical in the management of CML [16,17].

The quantification of *BCR-ABL1* transcripts and the detection of BCR-ABL1 kinase domain (KD) mutations enable timely therapy switches and selection of the most appropriate treatments [18]. Although multiple targeted therapies are available for CML patients, it is challenging to select the best targeted therapy to each patient. Therefore, the use of intelligent techniques (e.g., artificial intelligence (AI)) [19] and the development of mathematical modeling and computational prediction methods could anticipate the underlying mechanisms of drug resistance and facilitate the design of more effective treatment strategies to improve drug efficacy [20].

## 2. Molecular Mechanisms

**Figure 1:**
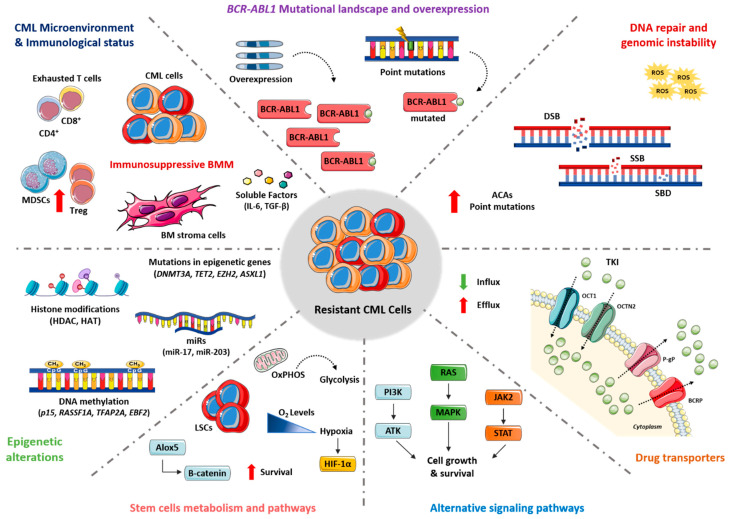
Molecular mechanisms of resistance to TKIs in CML. The molecular mechanisms responsible for TKI resistance in CML include: BCR-ABL1 mutations and BCR-ABL1 overexpression; alteration of DNA damage repair and genomic instability (increasing the additional chromosome abnormalities (ACAs) and point mutations); changes in drug transporters activity (e.g., increased efflux and decreased influx); activation of alternative signaling pathways (e.g., PI3K/AKT, JAK/STAT, and RAS/MAPK); changes in leukemia stem-cell metabolism and pathways (e.g., metabolic shift, Hypoxia/HIF-1α, and Alox5/β-catenin); epigenetic alterations (e.g., mutations on epigenetic regulating genes such as DNMT3A and/or increased methylation of p15 and EBF2 genes); altered expression of microRNAs (e.g., miR-17 and miR-203); changes in the microenvironment and immunological status (e.g., immunosuppressive bone marrow microenvironment (BMM) with increased MDSCs and Treg, plus exhausted T cells).

Several mechanisms are associated with TKI resistance, including *BCR-ABL1* mutations and overexpression, abnormal activity of drug transporters, activation of alternative signaling pathways, DNA repair, and genomic instability, epigenetic dysfunction, leukemia stem cell (LSC) persistence, and dysfunction of the immune system (Figure 1).

### 2.1. BCR-ABL1 Mutational Landscape and Overexpression

The effectiveness of TKI treatment is highly dependent on proper BCR-ABL1–drug interaction [21], and the most studied mechanisms are those responsible for the reactivation of BCR-ABL1 kinase activity [22]. Overexpression of *BCR-ABL1* and mutations on the fusion gene that impair the binding of TKI to targeted kinase may lead to resistance and are classified as *BCR-ABL1* dependent mechanisms [23].

The occurrence of point mutations on the *ABL1* KD is the most common TKI resistance mechanism, being more frequent in acquired resistance rather than primary resistance cases and is associated with poor prognosis and higher risk of disease progression [13,24,25]. Over 100 different mutations have been identified, affecting more than 50 amino acids [26]. The mechanism of action of these mutations includes decrease affinity of TKI to the binding domain or changes in BCR-ABL1 conformation [27]. The frequency of mutations increases with disease progression, occurring in approximately 75% of myeloid CML-blast crises (-BC) cases [14]. The mutations’ appearance could result from genetic instability induced by BCR-ABL1, by the selective pressure of pre-existing mutant clones, and/or the drug itself, which gradually outgrow the drug-sensitive cells [28,29].

The first described *BCR-ABL1* mutation was T315I (isoleucine replaces threonine in position 315 of BCR-ABL1), a mutation in the TKI binding site. This is the most frequently detected mutation among resistant patients (frequency between 4 and 20%) [26,30]. T315I, called a “gatekeeper” mutation, confers resistance to all TKI approved to frontline being only sensitive to Ponatinib [31]. The location of mutations has different impacts on TKI treatment effectiveness, with variable degrees of sensitivity to the different TKIs (Table 1). *BCR-ABL1* point mutations can be classified into five categories: (I) mutations in the P-loop (ATP-binding site); (II) mutations that directly affect the binding of TKI (drug contact site); (III) mutation in the catalytic domain (C-loop); (IV) mutations on the activation (A)-loop; (V) mutations in myristate pocket [27,32]. Mutations on the P-loop have been the most commonly observed in resistant patients, representing 36 to 48% of cases, alongside T351I [33]. These mutations induce destabilization of BCR-ABL1 conformation, impairing Imatinib association as well as Nilotinib action. Additionally, patients carrying P-loop mutations are related to a higher risk of disease progression [33].

The knowledge of this resistant mechanism has justified the development of second and third-generation TKIs, able to overcome and inhibit mutated *BCR-ABL1*. However, even with these new-generation TKIs, some mutations, such as T315I, remain resistant to multiple TKIs [15]. With the approval of second-generation TKIs as frontline CML treatment, fewer mutant clones are expected compared to those emerging under Imatinib treatment since these TKIs can bind even in the presence of some mutations [36]. The majority of these *BCR-ABL1* mutants are resistant to at least one single-agent TKI, but Eide et al. (2019) proposed that combination treatment in particular with Ponatinib and Asciminib can be a strategy to overcome this type of resistance [37]. According to the 2017 European Society for Medical Oncology (ESMO) management guidelines, *BCR-ABL1* mutational analysis is recommended for patients who fail Imatinib or second-generation TKIs and those who progress to accelerated phase (AP) or BC [38]. However, 30–40% of patients with suboptimal responses harbor low-level resistance mutant clones that are detected by Sanger sequencing and will be selected unless therapy is changed [39]. In this context, a sensitive screening approach should be included in the clinical algorithms for patients with suboptimal responses.

Nevertheless, the presence of one mutation on *BCR-ABL1* is not exclusive, as the occurrence of other mutations in the same sequence is possible [27]. In some patients, multiple mutations are identified in different BCR-ABL1 molecules (different CML clones), and this is called a polyclonal mutation. However, a current issue in CML treatment is the compound mutations which are the presence of two or more mutation occurring in the same BCR-ABL1 clone [27]. Although individual mutations could be sensitive to a TKI, the interaction between them could lead to resistance [40,41]. One example is the compound mutation T315I/E255V. Each of these mutations, when isolated, are responsive to Ponatinib; however, when together, they exhibit increased resistance to this third-generation TKI [41].

The occurrence of mutations outside the kinase domain is less frequent but could impact on TKIs resistance. Mutations on the SH domain could affect the BCR-ABL1 conformation and consequently compromise the TKI efficacy [42,43]. In addition to point mutations, some studies have reported resistance acquisition by abnormal splicing of *BCR-ABL1*. These rare splicing mutations are associated with nucleotides insertion, namely the retention of 35 intronic nucleotides on exon 8 to 9 of *ABL1* [44,45]. Furthermore, some mutations that confer resistance to Asciminib have been identified (Table 1), and available preclinical and clinical studies have suggested that mutations in and around the myristylation pocket may also confer resistance to this TKI [32,37,46].

*BCR-ABL1* expression due to gene amplification or upregulation at the transcriptional level is another resistance mechanism that is only observed in a small proportion of patients [30,47]. The overexpression of *BCR-ABL1* leads to resistance by increasing the oncoprotein concentration needed to be inhibited with TKI. Besides being more probably to occur, amplification is less frequent than point mutations [28], and in clinical settings, this resistance mechanism is associated with increased *BCR-ABL1* transcription [22]. Some authors hypothesized that this amplification or overexpression of *BCR-ABL1* precedes the emergence of point mutations in the kinase domain [29].

### 2.2. DNA Damage Repair and Genomic Instability

The DNA damage response (DDR) deregulation that leads to DNA damage and genomic instability has been implicated in the CML evolution, leading to TKI resistance and disease transition from CP-CML to more malignant stages [48,49,50]. This fact is supported by the higher frequency of copy number alterations and numerical and structural chromosomal changes observed in CML patients in BC compared with those in CP—a sign of increasing genomic instability [49]. Genetic instability is also a common feature in TKI-refractory CML patients [50]. The presence of 3q26.2 abnormalities, a minor route for additional chromosomal abnormalities (ACAs), are associated with TKI resistance and poor prognosis [51]. The frequency of ACAs increases from 5 to 7% in CML patients in CP up to 70 to 80% in BC and around 17% in TKI resistant patients, emphasizing the role of DDR dysregulation in the CML course and TKI resistance [52,53,54].

The BCR-ABL1 oncoprotein is responsible for the genomic instability observed in CML since its activity generates reactive oxygen species (ROS), disrupts the DDR pathways activating error-prone DNA repair, induces replication stress and centrosomal dysfunction, and inhibits apoptosis resulting from different DNA damage-induced lesions [50,55,56]. Although the activation of DNA damage repair pathways is increased to counteract DNA damage, unfaithful and error-prone pathways such as alternative non-homologous end joining (NHEJ), single-strand annealing (SSA), and unfaithful homologous recombination repair (HRR) are enhanced in Ph-positive cells [57,58,59,60,61,62]. In these cells, the usually faithful HRR induces point mutations, the NHEJ promotes extensive nucleobase loss, and the high activity of SSA generates large deletions [54]. Additionally, other DNA damage repair pathways are inhibited, including mismatch repair (MMR) and base excision repair (BER) [63,64,65,66,67], while the mutagenic nucleotide excision repair (NER) is promoted [68].

Some studies performed in cell lines demonstrated the involvement of DDR in TKI resistance. The upregulation of the alternative NHEJ factors PARP1, WRN, and DNA ligase IIIa along with the downregulation of the canonical NHEJ proteins Artemis and DNA ligase IV reflect the role of the inefficient and error-prone alternative NHEJ pathway in TKI resistance [69,70]. Furthermore, different in vitro models of TKI resistance showed upregulation of the BER genes *MBD4* and *NTHL1* [71,72]. Similarly, studies comparing sensitive and resistant Imatinib CML patients demonstrated that patients resistant to therapy have higher expression levels of DNA damage repair genes such as *RAD51L1*, *FANCA*, and *ERCC5* [73,74,75]. These facts support that DNA damage repair impairment in CML is directly involved in TKIs resistance and CML evolution. Importantly, the dysregulation of these mechanisms can also contribute indirectly to resistance through genetic instability and the consequent accumulation of point mutations and chromosomal aberrations. These point mutations can occur in the ABL1 kinase domain preventing the binding of TKIs. Moreover, point mutations and chromosomal aberrations can lead to the activation of alternative cellular signaling pathways that also contribute to TKI resistance, such as PI3K/AKT, JAK/STAT, RAS/MAPK, and SRC pathways [18].

### 2.3. Drug Transporters

Treatment efficacy is highly dependent on the access of the drug to its molecular target. For targeting BCR-ABL1 (a non-receptor tyrosine kinase), it is critical for TKIs to reach the inside of CML cells at adequate pharmacological concentrations to achieve therapeutic clinical outcomes. The movement of drugs across cell membranes is largely mediated by drug transporters proteins [76]. The balance between drug influx and drug efflux is crucial to BCR-ABL1 inhibition by TKIs, and changes on these transporters may explain the resistance phenotypes caused by ineffective TKI uptake and/or excessive extrusion of TKI from the cell [76,77].

Most drugs have a low ability to diffuse freely across cell membranes, as their movement is not dependent on ATP and is mediated by solute carrier (SLC) transporters, such as OCT1 [78]. OCT1 is the main drug transporter responsible for the TKI uptake, and its expression or activity impacts drug response levels [76]. This protein is encoded by the *SLC22A1* gene, and higher mRNA levels were associated with major molecular responses [79,80]. On the contrary, low OCT1 expression is a common trait in multidrug resistance and is associated with suboptimal responses [81,82]. Some authors have identified the functional activity of OCT1 in leukemic cells at diagnosis as a prognostic marker of TKI response [83]. However, the results obtained regarding OCT1 expression and activity are controversial since many authors fail to observe a significant correlation with Imatinib transport or response [84]. Other transporters have been identified as mediators of TKI transport, namely OCTN2, OATPs, and MATE1 [78,85,86]. Harrach et al. proposed that measurement of MATE1 expression levels before treatment can help in identifying Imatinib non-responder patients [86]. In Imatinib-resistant cell lines, Alves et al. observed a parallel decrease in OCT1 and OCTN2 expression showing the contribution of more than one influx transporters to the resistance process [87]. Lower levels of TKI uptake could be overcome with a switch to Dasatinib since this TKI can cross the cell membrane by diffusion [88,89].

Extrusion of metabolites, xenobiotics, and chemotherapeutic agents is mediated by ATP-binding cassette (ABC) transporters [77,90]. Overexpression of these transporters reduces the intracellular drug concentration, affecting its effectiveness [91]. P-glycoprotein (P-gp) is the most studied transporter, and its overexpression has been described in several chemo-resistant cancers [76]. All TKIs approved for CML treatment are recognized substrates of P-gp [92]. High *ABCB1* expression levels (the gene that encodes P-gp) are associated with poor long-term outcomes and advanced-phase disease [18,93,94]. According to Eadie et al., in the dynamic process of resistance acquisition, the P-gp overexpression may work as an initiator process that favors the development of another mechanism of resistance [95]. Another essential transporter for TKI resistance is the breast cancer resistance protein (BCRP), codified by the *ABCG2* gene [96]. This protein is present in stem cells, and its function is particularly relevant on LSCs, protecting them from TKI action [97,98]. As described for P-gp, high levels of BCRP are associated with resistance, while low rates are correlated with molecular response [99,100]. Additionally, other ABC transporters may be involved in TKI extrusion as MRP6 (*ABCC6*), which share some substrates with P-gp. The MRP6 may be especially relevant in resistance to second-generation TKI [101].

Genetic variants highly influence the function, expression, and localization of drug transporters. The presence of polymorphic variants in genes actively involved in TKI transport may influence its pharmacokinetics and, consequently, drug efficacy [77,102]. For *ABCG2*, rs2231142 results in loss of function mainly by altering protein folding, and a significant reduction of the transporter has been linked to the presence of the A allele [103,104,105]. Many groups found an association of the A allele with higher molecular response rates, while the CC genotype was correlated with TKI resistance [106,107,108]. Moreover, the G allele in rs683369 of *SLC22A1* has been previously associated with lower major molecular responses (MMR) and high risk of resistance due to low expression of OCT1 and consequent lower TKI uptake [107,109,110]. Thus, lower drug uptake or high drug extrusion may create a favorable environment for CML cells acquiring other resistance mechanisms, such as *BCR-ABL1* mutations [23,111].

### 2.4. Alternative Signaling Pathways

**Figure 2:**
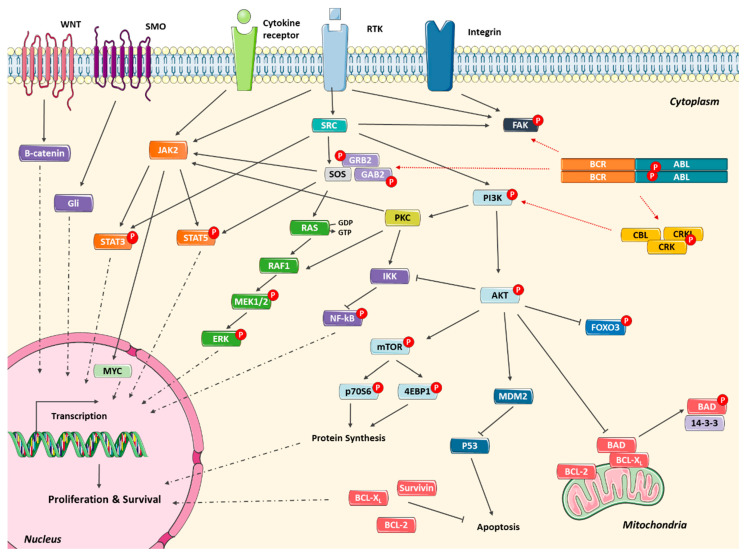
Alternative to BCR-ABL1 signaling network. To evade BCR-ABL1 inhibition, CML cells activate alternative signaling pathways including RAS/MAPK, SRC, JAK/STAT, WNT/b-catenin, hedgehog, and PI3K/AKT. The transduction of oncogenic signals culminates with the activation of multiple downstream signaling pathways that enhance survival, inhibit of apoptosis, and alter cell adhesion and migration. A subset of these pathways and their constituent transcription factors (β-catenin, Gli, STAT5, MYC, FOXO3), serine/threonine-specific kinases (RAS/MAPKs, PI3K/AKT/mTOR), and apoptosis-related proteins (BAD, BCL-2, BCL-XL, survivin) are shown. It is important to note that this is a simplified diagram and that many more associations between BCR-ABL1 and signaling proteins have been reported.

To overcome the inhibition of BCR-ABL1, CML cells may activate alternative signaling pathways to compensate the loss of BCR-ABL1 kinase activity. Consequently, cells will be able to proliferate and survive despite effective BCR-ABL1 inhibition. RAS/MAPK, SRC, JAK/STAT, and PI3K/AKT are some of the pathways that contribute to TKI resistance (Figure 2).

GAB2 is one member of the GAB family docking proteins that exerts a critical role in CML by amplifying BCR-ABL signaling [112]. Dysregulation of this protein results in increased proliferation, reduced growth factor requirements, and enhanced cellular motility [112]. Additionally, persistent phosphorylation of GAB2 results in activation of substrates such as RAS protein that stabilizes in the active form after GAB2 activation [113,114]. The increased expression of protein kinase C (PKC) was also observed in TKI-resistant CML cells [115]. In a recent study, Ma and collaborators demonstrated that PKC-β overexpression was associated with resistance to TKIs and its inhibition in CML CD34^+^ cells increased the sensitivity to Imatinib [115].

Overexpression of the SRC family kinase protein, such as LYN and HCK, are related to CML resistance cases [5,116]. Therefore, the activation of SRC function is crucial for cell proliferation, survival, and adhesion, and is a compensatory mechanism in the case of BCR-ABL1 inhibition [117]. These SRC proteins lead to AKT activation promoting survival and STAT5 activation stimulating proliferation [118]. The overexpression of SRC proteins in CML was the rationale for development and use of dual SRC/ABL inhibitors, such as Dasatinib and Bosutinib [5]. Additionally, STAT signaling can be activated through the JAK2 protein. In response to cytokines released by cancer cells and bone marrow niche cells, JAK2 is activated and subsequently phosphorylate one of the seven STAT members [119]. STAT3 and STAT5 have been identified as the most relevant STAT proteins in cancer [119]. After STAT phosphorylation, this protein migrates to the nucleus regulating the transcription of various target genes, e.g., c-MYC. In CML, STAT5 is involved in disease progression, promoting cell cycle progression and ROS production, inhibiting apoptosis, and up-regulating P-gp expression [120]. Due to its pleiotropic effects, low levels of STAT5 have been correlated with TKI sensitivity. On the other hand, STAT3 emerges as a critical molecule in the resistant phenotype, including TKI resistance [121]. Phosphorylation of STAT3 on residue 727 and STAT5 in residue 694 is reduced under treatment with TKIs, but the phosphorylation on residue 705 of STAT3 is not altered [122].

After activation of PI3K, AKT is subsequently phosphorylated, influencing multiples downstream proteins. BAD is one of AKT targets that reduces the apoptotic signal. After being phosphorylated, BAD becomes inactive and consequently did not inhibit anti-apoptotic proteins like BCL-2 and BCL-XL [123]. Another AKT target is the FOXO transcription factors, which under normal conditions regulate cell cycle arrest and apoptosis. The AKT induced phosphorylation of FOXO blocks its activity avoiding apoptosis and promoting cell cycle progression [124]. Furthermore, mTOR is a serine/threonine kinase that is activated by AKT and regulates mRNA translation, controlling cell growth and proliferation [125]. In the same way, NF-kB is also indirectly activated by AKT, promoting gene transcription. AKT targets IKK, the natural inhibitor of NF-kB, releasing this repression signal from NF-kB that can then translocate into the nucleus acting as a transcription factor [126].

Furthermore, CML patients resistant to TKIs and Imatinib-resistant cell lines show higher survivin levels than the sensitive ones [127]. Survivin is an inhibitor of apoptosis protein (IAP), downstream of the BCR-ABL1 signaling pathway, known to regulate the cell cycle and apoptosis, favoring the survival of cancer cells by evading cell death and promoting cell division [128]. Zhou et al. showed that a combination treatment of a WNT/β-catenin signaling inhibitor with Nilotinib synergistically killed KBM5^T315I^ cells (a CML resistant cell line) as well as primary BC-CML cells obtained from TKI-resistant patients (with and without BCR-ABL1 kinase mutations) by decreasing the expression of CD44, MYC, p-CRKL, p-STAT5, and survivin [129].

Additionally, all signaling pathways mentioned along with the BCR-ABL1 activity itself lead to an accumulation of ROS in CML cells [130,131]. Some studies reported in primary BCR-ABL1-positive cells up to six times more ROS levels than in normal cells [132]. This oxidative cellular milieu contributes to a higher genetic instability potentially leading to an increased probability of point mutations [70,133]. The unsatisfactory response rates to TKIs and therapy failure observed in some patients can occur due to mutations downstream of BCR-ABL1 or in compensatory alternative signaling pathways (see above) [24]. In CML-BC patients, several additional genetic abnormalities have been detected. These abnormalities (among others) include: (I) mutations: *IKZF1*, *RUNX1*, *ASXL1*, *BCORL1*, and *IDH1*/*IDH2*; (II) fusions: *MLL*, *MSI2*, and *MECOM*; (III) deletions: *PAX5/CDKN2A*, *HBS1L-MYB*, and del(17p); (IV) amplifications: chromosome 8, 19, and 17q [134,135]. These mutations, along with epigenetic reprogramming, facilitate the BCR-ABL1 independent activation of PI3K, MAPK, JAK/STAT, and SRC signaling pathways in CML cells, all of which have been implicated in BCR-ABL1 independent mechanisms of resistance [22]. However, the detection of the signaling pathway responsible for the resistant phenotype is in many cases difficult, hampering the discovery of a suitable target to use in combination with TKI to circumvent resistance.

The impairment of multiple signaling pathways induced by BCR-ABL1 or by independent mechanisms results in a favorable cancer environment, contributing to poor TKI efficacy and consequently to resistance. The knowledge of this network supports the rationale to new therapeutic schemes with other inhibitors or in combination with TKIs.

### 2.5. Stem Cell Metabolism and Pathways

The LSCs display high resistance to TKI showing heterogenous adaptations including a modified transcriptome, genome, and epigenome [136]. Given the extensive divergence of the numerous BCR-ABL oncogene-independent pathways that are deregulated in LSCs, it is not surprising that cellular metabolic reprogramming (an emerging hallmark for cancer stem-cell biology) [137] has been also implicated in LSC survival adaptations and resistance to TKI treatment. Specifically, while normal cells mostly exploit glucose for producing energy via mitochondrial oxidative phosphorylation (OxPHOS), cancer cells may switch to increased rates of glucose uptake and aerobic glycolysis, a process also known as the Warburg effect [138]. OxPHOS is critical for energy production as well as for supplementation of anabolic precursors in LSCs [139], representing thus a vulnerability that can be targeted by selective therapeutics. On the other hand, the low O_2_ tension (hypoxia) that characterize the bone marrow microenvironment (BMM) niches stabilizes hypoxia-inducible factor 1α (HIF-1α) which is a crucial regulator of maintenance, survival, and proliferation of LSCs [140].

The BCR-ABL1-mediated activation of the nutrient-sensitive pathways leads to GSK3-β suppression along with the cytosolic retention and degradation of FOXOs, a number of pleiotropic transcription factors that (among others) are activators of autophagy [141]. Upon treatment with TKIs the PI3K/AKT signaling is blunted in CML (including LSCs) leading to inhibition of the pro-survival β-catenin signaling [142]; yet it also enables activation of FOXOs, likely offering to LSCs a BCR-ABL1-independent route for survival. In support, FOXO signaling maintains LSCs in a CML-like myeloproliferative disease mouse model [143]. The autophagic process also generates adenosine triphosphate (ATP) and essential building blocks (e.g., amino acids) during oxygen and/or nutrient deprivation [144]. Thus, it is not surprising that it essentially helps tumor cells (including LCSs) to tolerate metabolic stress (e.g., triggered by TKIs) [145] and/or suppress apoptosis induced by anti-tumor agents [146]. Interestingly, basal autophagy is higher in CML-LSCs as compared to normal HSCs and it is further upregulated following treatment with TKIs. As expected, inhibition of autophagy enhances the selective anti-tumor activity of tigecycline to overcome drug resistance in CML [147], and effective inhibition of autophagy using second generation autophagic inhibitors potentiates TKI-induced cell death of LSCs [148].

Metabolomics studies have shown that LSCs accumulate high levels of various dipeptides consisting of a range of amino acids [149]. Given the LSCs’ need for essential building blocks, the internalization of dipeptides and oligopeptides is an energy-saving process that supplies intracellular amino acids. This mechanism is related to the upregulation of the oligopeptide/dipeptide transporter SLC15A2 which, by supplying dipeptides, activates the p38/MAPK-Smad3-FoxO3a axis [149]. Similarly, the branched-chain amino acid (BCAA) valine is central to HSC self-renewal capacity [150] and the BCAA levels were significantly increased in these cells in a CML-BC mouse model. This increase is due to the upregulated expression and activity of the BCAA amino acid transaminase 1 (BCAT1) [151]. Notably, increased metabolism of BCAA activates mTORC1 that acts as a pro-survival pathway of BC-CML-initiating cells [151].

Regarding regulation of the tricarboxylic acid (TCA) cycle and OxPHOS, a comparative gene expression analysis in CML stem-progenitor cells isolated from TKI-responding and non-responding patients revealed upregulation of *ILK* (integrin-linked kinase) in LSCs of non-responding patients [152]. It was shown that ILK regulates quiescence of non-responding LSCs through the OxPHOS pathway [152]. The multifunctional sirtuin 1 (SIRT1) was also found to be induced in CML-LSCs and contributes to their resistance to TKIs [153]. The upregulated mitochondrial OxPHOS is an important survival mechanism of CML-LSCs [139]. Supportively, deficiency of the *Sirt1* gene downregulated OxPHOS-related mRNAs in LCSs and delayed CML onset and progression in a mouse CML model [154]. Mechanistically, it was found that loss of SIRT1 reduced PGC-1α acetylation resulting in suppressed OxPHOS activity [154]. Further to these findings it was shown that targeting mitochondrial OxPHOS via tigecycline suppressed the mitochondrial respiration and the proliferative capacity of therapy-resistant CML-LSCs [139]. Moreover, analysis of patient-derived LSCs revealed increased aerobic ATP production which correlated with high expression and activity levels of pyruvate carboxylase [139]. *Hif-1a* knockout in a mouse model suggested that Hif-1a enhanced glycolysis and possibly contributes to CML stem-cell survival [140].

Lipids and fatty acid metabolism are also involved in LSCs survival, since arachidonate 5-lipoxygenase (encoded by arachidonate 5-oxygenase, Alox5) was found to be upregulated in LSCs and modulate β-catenin levels in a BCR-ABL1-independent manner [155,156]. Interestingly, both Alox5 and arachidonate 15-oxygenase (Alox15) are overexpressed in CML stem cells and are not suppressed by TKIs [156,157]. Moreover, loss of the Alox5 gene impaired LSCs and prevented CML development [156] while activation of the PKC-β/Alox5 axis promoted BCR-ABL1-independent TKIs resistance in CML [115]. Alox5- or Alox15-deficiency in mice resulted in decreased self-renewal capacity of CML stem cells as well as in reduced rates of CML onset [156,157].

### 2.6. Epigenetic Alterations

There is now ample evidence that epigenetic dysregulation contributes to leukemic stem cell generation, maintenance, and progression in CML. Several studies have demonstrated that mutations in epigenetic regulating genes, such as *DNMT3A* [158], *TET2* [159], *EZH2* [160], and *ASXL1* [160], are relatively uncommon in chronic phase CML [160] but the incidence of these mutations increase during disease progression [161,162].

Epigenetic modifications consist in the addition or removal of small molecules, such as methyl or acetyl groups, onto DNA or DNA-related proteins, such as histones, resulting in the remodeling of chromatin and providing sites for the recruitment of other transcription factors [163]. In addition to the modification in nuclear molecules, post-translational modifications may also have a significant effect on the phenotype of CML and its responsiveness to therapy [164]. DNA hypermethylation is a common oncogenic process in many solid and hematological tumors. This has been well documented in CML patients, especially those with AP and BC [160,165]. Although *ABL-1* hypermethylation has been well documented, its role in the pathophysiology of disease progression is not clear. It is, however, evident that increased methylation of genes such as *p15* [166], *RASSF1A* [167], *TFAP2A* [168], and *EBF2* [168], among others, is a frequent event in disease progression [169,170]. This is in line with what is observed in myelodysplastic syndrome (MDS) and acute myeloid leukemia (AML), especially secondary AML and AML with MDS-related changes [171].

In parallel to DNA hypermethylation, malignant tissues also acquire histone modifications. The most frequent of these are acetylation, methylation, and phosphorylation. Their main effect is the modulation of chromatin condensation which subsequently alters expression of cell cycle, apoptotic, and tumor suppressor genes [172]. Specific enzymes regulate these processes and the histone acetyl-transferases (HAT) and histone deacetylases (HDAC) are the best studied ones. In general, deacetylation of histones leads to silencing of genes in the affected locus. Increased expression of HDAC has been documented in several malignant cells, including CML, resulting in loss of the tumor suppressor scaffold/matrix attachment region binding protein 1 (SMAR1) [173], which in turn increases cyclin D1 expression, and suppresses p53 [174], impeding its regulation of the cell cycle.

Recent research has uncovered that, in addition to epigenetic regulation at a nuclear level, post-translational processes play a critical role in epigenetic regulation of protein synthesis. MicroRNAs are the main mediators of these processes, exerting their effect by blocking protein synthesis and promoting mRNA degradation [164]. CML patients exhibit clearly distinct microRNA expression patterns compared to healthy individuals and patterns of microRNA expression also differ between CML patients in different phases of the disease and between those that do and do not respond to TKI [175]. One such example is miR-150, which is downregulated in CML patients at diagnosis as compared to healthy individuals and in advanced phases of disease as compared to CP [176,177]. Interestingly, patients who respond to TKI increase miR-150 levels to levels seen in the normal population. Similar findings were observed for miR-146a [178] and miR-10a [179], with reduced levels seen at diagnosis and in advanced phases and normal levels in patients who respond to TKI. In Imatinib-resistant CML K562 cells (K562-RC and K562-RD cells), the oncomiRs miR-21 and miR-26b were upregulated and the tumor suppressor miR-451 was downregulated in comparison with sensitive cells [180]. Other mi-RNAs, such as miR-19a, miR-19b, miR-17, miR-130, and miR-150, are increased in CML [181,182]. The expression of some of these miRs is directly regulated by BCR-ABL1 through its effect on miR effectors, such as *MYC* in the case of miR-17 [183], *CCN3* in the case of miR-130 [184], and *MYB* in the case of miR-150 [176]. Some miRs may also interact directly with BCR-ABL1, like miR-203 [185], which has an inhibitory effect on BCR-ABL1 and expression is suppressed in CML through hypermethylation.

### 2.7. CML Microenvironment and Immunological Status

BMM is considered a safe haven for HSCs [186,187] and in CML, as described in other neoplasias, the leukemic cells become progressively independent of physiological control of BMM [188,189,190]. In addition, the leukemic cells shape the phenotype and function of surrounding cells, reprograming the BMM to a more favorable environment for leukemic cell survival, proliferation, immune escape, and drug resistance [191]. This type of resistance could be mediated by changes in direct cell–cell contact, production/secretion of cytokines and growth factors, and/or the establishment of a hypoxic environment [192].

The bidirectional interaction between CML cells and the BMM niche is vital to support leukemic development and counteract the TKI effects [187,190]. Alteration in cell adhesion to stroma may provide chemoprotection to CML cells [193]. Kumar et al. (2020) showed that BCR-ABL1 T315I mutated cells presented alteration in the actin cytoskeleton, integrin β3 levels, in the expression and phosphorylation levels of FAK and ILK, and fibronectin expression when compared with BCR-ABL1 sensitive cells [194]. In CML, the increased expression of integrin β3 and ILK interaction with β integrins was associated with Imatinib resistance through the activation of PI3K/AKT, STAT3, and ERK1/2 signaling pathways [152]. Furthermore, the interaction with MSCs can also be mediated by N-cadherin and the activation of β-catenin signaling, which are crucial for LSC [195]. Dysregulation of cytokines, growth factors, and their receptors contribute to CML protective environment, leading to CML persistence and resistance to treatment [196,197]. These factors are partly due to the BMM component’s activity, but also to CML cells through autocrine and paracrine signaling [196,197]. CXCL12, IL-3, VEGF, FGF, TNF-α, IL-6, IL-7, and TGF-β, among others, are cytokines and growth factors altered in CML [198]. Studies have shown that CD34^+^ cells from patients with CML produce ten times more cytokines than their normal counterpart, highlighting the importance of these signaling molecules to this disease [198]. TKI treatment can reverse the levels of these soluble factors albeit not in a complete manner. Signaling from different cytokines, including of IL-7, acts through the JAK2/STAT pathway to balance apoptosis induced by TKI [119,199]. This culminates in sustained activation of STAT3, with increased expression of anti-apoptotic genes, e.g., *BLC-2*, *BLC-XL*, and *MCL-1* [121]. CXCL12 signaling pathway is crucial for the maintenance of healthy HSCs, but, in CML BMM the levels of CXCL12 are reduced. This promotes the expansion of CML stem cells by increasing their self-renewal capacities [197,200]. Depending on the factors released, multiple pathways could be activated to avoid TKI effect. Another important pathway of cell communication is through microvesicles release, which can transport different mRNAs, miRs, and proteins. One example is the communication of CML cells with HSCs. Through miR-146b transfer, microvesicles derived from the CML cell line K562 induced the transformation of HSCs into leukemic cells [201]. Similarly, CML cells interact with MSCs by increasing TGF-β1 in MSCs, which causes TGF-β1-dependent proliferation of *BCR-ABL*-positive cells as a feedback loop [202]. Furthermore, the hypoxic environment may also have implications for drug resistance. Even with BCR-ABL1 inhibition, hypoxic conditions of BMM induce CML cells survival through the activation of HIF-1 signaling pathway [27,199].

The immune system is an essential player within the BMM, and expression of specific immune cells might dictate successful TKI responses [203,204]. At diagnosis, CML is characterized by immune dysfunction, with a reduction in the number and function of NK and dendritic cells as well as dysfunctional CD8+ cytotoxic T cells [203,205]. Associated with these abnormalities, myeloid-derived suppressor cells (MDSCs) and regulatory T cells (Tregs) are increased, contributing to T-cell dysfunction. After TKI therapy, the levels of immune cells are restored to normal levels [203]. However, during disease progression and drug resistance, CML cells adopt strategies to escape immunosurveillance [191,206]. One example is the aberrant expression of immune checkpoint, such as molecules in the PD-1/PDL-1 axis, which has been associated with immune evasion. Overexpressed PDL-1 on tumor cells will function as a co-inhibitory molecule for T cells (expressing PD-1), leading to T-cell exhaustion and anergy [206,207,208]. The creation of an immunosuppressive and inflammatory BMM is crucial for LSC preservation and drug resistance. Leukemic cells induce the expansion/activation of MDSCs directly through the release of microvesicles that will reprogram MDSC and indirectly through MSCs, which overexpress immunomodulatory factors (such as TGF-β, IL-6, and IL-10) capable of activating MDSCs. This culminates into increased Treg levels, T-cell inhibition, and dysfunction of NK cells [202,209]. The relevance of BMM and immunological status in CML was highlighted in TFR studies, where specific immune cell types have been proposed as predictive biomarkers of successful TKI discontinuation [204,210,211].

## 3. Methodologies to Access TKI Resistance

CML treatment optimization has been achieved by the implementation and standardization of molecular monitoring strategies such as real-time quantitative reverse transcription polymerase chain reaction (RT-qPCR). The different methodologies used for CML diagnosis and treatment monitoring in the clinic have undeniably improved the effectivity of patients’ management, improved the detection of the BCR-ABL1 KD mutations, and refined the selection of CML patients with higher probability to achieve treatment-free remission (TFR) after TKI cessation.

### 3.1. Molecular Approaches

The *BCR-ABL1* gene is, at the same time, both a therapeutic target and a robust and precise biomarker of minimal residual disease (MRD). Over the years, CML treatment has been optimized through the implementation and standardization of molecular monitoring. According to the 2020 European Leukemia-Net (ELN) recommendations, the molecular testing of CML should include different molecular methodologies [212]. At diagnosis, it is recommended to perform cytogenetics (chromosome banding analysis) to detect the Ph chromosome, fluorescence in situ hybridization (FISH) in Ph-negative patients, and qualitative PCR to identify the *BCR-ABL1* fusion gene type. During treatment, the regular quantification of *BCR-ABL1* transcripts by quantitative real-time PCR is performed to monitor transcript levels. After the achievement of a complete cytogenetic response, cytogenetic tests are warranted only in cases with ACA in the Ph^+^ clone [212]. These recommendations also establish time-dependent molecular response (MR) milestones with prognostic significance, optimal response monitorization, and the foundations for treatment-free remission (TFR). Deep molecular response (DMR) is considered of crucial clinical importance to identify patients with a high probability to remain in remission after discontinuing TKI therapy [213]. In TFR, only quantitative PCR for *BCR-ABL1* transcripts is needed [214]. The 2020 ELN recommendations defined more rigorous TFR criteria demanding typical *BCR-ABL1* transcripts, a minimum of 4–5 years of TKI therapy, and a DMR of MR^4^ or better during more than 2 years [212]. The success of TKI discontinuation is mainly predicted by a durable DMR [215,216]. However, quantitative PCR might not be the best methodology to select CML patients for TKI discontinuation and for molecular monitoring during TFR, since the majority of patients (50–60%) with undetectable DMR by quantitative PCR eventually lose major molecular response (MMR) [215,217,218].

In clinical settings, some molecular tests have been robustly validated for detecting and monitoring *BCR-ABL1* transcripts [213]. The “classical” quantitative PCR (RT-qPCR) has several intrinsic limitations, including the detection limit of three copies of *BCR-ABL1* transcript, the need for a standard curve, and the sensitivity to inhibitors. However, several studies have tried to overcome these limitations by improving its performance [214]. Three recently FDA-approved tests seem to perform better than the standard RT-qPCR tests and are more attractive for monitoring very low levels of *BCR-ABL1* transcripts [213]. These tests are the QuantideX^®^ qPCR BCR-ABL IS Kit (Asuragen Inc., Austin, TX, USA), with a sensitivity of 0.002% IS (MR^4.7^) for CML patients expressing e13a2 or e14a2 fusion transcripts, the Xpert^®^ BCR-ABL Ultra (Cepheid, Sunnyvale, CA, USA), which detects the most common BCR-ABL transcripts below MR^4.5^ or 0.0032% with a short turnaround time, and the digital PCR (dPCR) kit QXDx BCR-ABL %IS (Bio-Rad Laboratories Inc., Hercules, CA, USA) [219,220,221]. This dPCR kit was compared with the gold standard RT-qPCR, and the assays were strongly correlated (*r* = 0.996) in the range between 20% to 0.002% [222]. These results supported the recommendation of dPCR as the standard of care for monitoring of patients with CML [214]. Moreover, a multicenter international study confirmed that dPCR is a valid alternative to RT-qPCR, showing a detection rate of 90.9% at MR^4.5^, 81.2% at MR^4.7^ (0.002% BCR-ABL1/ABL1 level), and 81% at MR^5.0^, with a low interlaboratory variation and high assay linearity [214,223,224]. These data indicate the usefulness of dPCR for MRD monitoring, particularly in CML patients with low *BCR-ABL1* levels and those potentially eligible for TKI discontinuation. However, to successfully implement dPCR in clinical settings, this methodology should be optimized and standardized to CML MRD monitoring.

Additionally, molecular testing is crucial in the setting of TKI therapy failure since these patients may have acquired *BCR-ABL1* point mutations that impair TKI binding [214]. The gold standard for *BCR-ABL1* KD mutation screening is Sanger sequencing, but this technique has relatively poor sensitivity (10–20%). The clonal configuration of *BCR-ABL1* mutations is very important since compound mutations are extremely resistant to first, second, and even third-generation TKIs in some cases [41,213,214,215]. In the context of *BCR-ABL1* point mutations, next-generation sequencing (NGS) and dPCR are being investigated as alternative methodologies to Sanger sequencing. The NGS approach has better sensitivity (1%) than Sanger and can distinguish between compound and polyclonal *BCR-ABL1* mutations when multiple substitutions fall on the same sequence reads. However, this methodology has a high error rate, particularly when sequencing mRNA, due to the use of the error-prone reverse transcriptase [213]. Recently, NGS was shown to detect emerging mutations and to predict high-risk transformation, highlighting the importance of low-level mutations—mutations with a variant allele frequency of 3–20%—in clinical settings [39,225]. The advantages of NGS to detect *BCR-ABL1* KD mutations resulted in its inclusion in position papers and the 2020 ELN recommendations [212,226,227]. As mentioned, the dPCR has also been explored as a complementary or even alternative strategy to detect *BCR-ABL1* KD mutations. Soverini and collaborators (2019) compared Sanger sequencing, NGS, and dPCR in CML patients with failure or warning responses to TKI therapy [228]. In this study, a multiplex single-tube assay was used to detect and quantify mutations conferring resistance to one or more second generation TKIs (T315I/A, F317L/V/I/C, Y253H, E255K, F359V/I/C, E255V, and V299L) and showed a very good concordance between dPCR and NGS, independently of mutation type and variant allele frequency, in samples positive for second-generation TKI-resistant mutations. However, NGS remains a better methodology to detect emerging mutations due to the high number of different mutations that can confer resistance to TKIs [214,228].

### 3.2. Bioinformatics and Artificial Intelligence as Methodologies to Decipher Mechanisms of Action or Resistance to TKIs in Leukemia

The use and application of machine learning methods for the diagnosis of common types and subtypes of leukemia has been very successful over the past decades, as reviewed by Sarah et al. [229]. One of the most comprehensive efforts was accomplished ten years ago using genome-wide expression profiling in the diagnosis and subclassification of many different types of leukemias [230]. Following this line of research, artificial intelligence (AI) and machine learning (ML) methods have been proven to be also very useful for integrating large-scale-*omics* data from cancer patients and for analyzing gene expression profiles in response to different drugs [231]. In this scenario, positive associations between gene expression and anticancer drug activity allowed the discovery of gene targets for the drugs tested [232]. Conversely, a negative association between gene expression and drug activity measured in these assays (for example, detecting high expression of a gene corresponding to decreased activity of a drug) indicated that such a gene/protein could be mediating resistance and low sensitivity to the drug. These associations have been found using ML by Lee et al. [233], who identified molecular markers for targeted treatment of AML. They also found that high expression of *GPR34* and *ADRBK2* genes (encoding two G protein-coupled receptor kinases) was correlated with a lower activity of Sunitinib (a multi-targeted receptor tyrosine kinase, RTK, inhibitor).

A recent study that also used ML models was able to predict future diagnosis of CML based on the analysis of data from retrospective electronic health records [234]. In particular, the ML models could predict CML using blood cell counts prior to diagnosis. These findings indicate that a ML model trained with blood cell counts can lead to diagnosis of CML earlier in the disease course as compared to usual medical care [234]. Other authors have recently developed a leukemia artificial intelligence program (LEAP) using the Extreme Gradient Boosting (XGBoost) decision tree method for the optimal treatment recommendation of tyrosine kinase inhibitors (TKIs) in patients with CML-CP. This work reports that the AI method consistently won international data analysis challenges selecting the optimal frontline TKI with accurate prediction [235]. These recent examples show that the development of ML algorithms outperforms conventional statistical models in prediction accuracy, paving the way for a new era of personalized treatment recommendations for cancer patients.

## 4. Therapeutic Approaches against Resistance

During the treatment of a patient with CML, changes in therapeutic protocol may be due to several reasons: resistance, intolerance, or suboptimal response rates (warning criteria), causing a mandatory TKI switch in case of resistance. The selection of the best second-line therapy needs to be adjusted to patients’ characteristics, comorbidities, and toxicity of first-line TKI, among other factors [212]. Different strategies can be adopted to overcome the resistant phenotype and reestablish response rates, or even in some cases, to improve the probability of treatment-free survival. From BCR-ABL1 targeted therapies to other signaling pathway inhibitors or immunotherapies, multiple options have been explored in resistant CML not only in monotherapy but also in combination strategies (Table 2).

### 4.1. BCR-ABL1 Targeted Therapies

All TKIs approved for CML treatment are orally administrated and competitively inhibit BCR-ABL1 TK by binding at the ATP-binding site. Dasatinib, Nilotinib, Bosutinib, and Radotinib are second-generation TKIs, where Ponatinib is a third-generation TKI.

According to ELN guidelines, in case of resistance, the BCR-ABL1 KD mutation profile needs to be investigated to guide selecting the second line of treatment. Each TKI presents a different sensitivity profile to the different mutations identified (Table 1), where the first designed TKI (Imatinib) presents less potency in case of resistance [27,34,35]. All the approved next-generation TKIs were designed aiming reverse resistance and intolerance observed in patients treated with Imatinib, especially the point mutations identified as a critical factor for TKI efficacy. Dasatinib can bind to BCR-ABL1 in active and inactive conformation. This dual SRC/ABL1 inhibitor in vitro showed over 300-fold more potency than Imatinib and can also inhibit SRC family kinases [236], being recommended in the case of Y253H, E255V/K, and F359V/I/C mutations [212]. However, the toxicity profile of Dasatinib, particularly associated with pleural effusion and pulmonary hypertension, needs to be considered according to patient characteristics [237]. In opposition, Nilotinib results from a chemical modification of Imatinib, and in vitro has approximately 30-fold higher potency than first-generation TKI. Nilotinib binds to inactive conformation of BCR-ABL1, like Imatinib, but also targets PDGFR and c-KIT [238].

The resistance pattern to Nilotinib is very similar to that observed for Imatinib regarding drug transporters, but this second-generation TKI presented a different resistance profile to *BCR-ABL1* point mutations. Nilotinib is resistant to Y253H/F, E255K/V, and T315I [27,34,35,93] but is an alternative for second line treatment in cases of other mutations. Very similar to Nilotinib, the Korean approved TKI (Radotinib) presented an in vitro IC_50_ of 34 nM [41,239]. Nevertheless, cases of Radotinib resistance have been associated with *BCR-ABL1* point mutations, namely Y235H, E255V, T315I, and T315M [239] (Table 1). Bosutinib, a second-generation TKI, is a dual SRC/ABL1 kinase inhibitor that binds to BCR-ABL1 in both conformations, as described for Dasatinib [240,241]. In terms of recommendations as to which TKI should be used in the case of BCR-ABL1 resistance mutations, Bosutinib works for all identified mutations with the exceptions of V299L and T315I BCR-ABL1 point mutations [27,34,35] (Table 1).

The most aggressive point mutation identified in *BCR-ABL1* is the T315I, and Ponatinib was designed to overcome this mutation. Ponatinib is the only TKI recommend by the European LeukeniaNet for the T315I mutation and can also be used in F317L/V/I/C, T315A, and V299L. In vitro, Ponatinib presented 500 times more potency than Imatinib and binds to the inactive conformation of BCR-ABL1 [242]. Nevertheless, resistance to this third-generation TKI has been associated with compound mutation of *BCR-ABL1*, even those including T315I, and with influx and efflux drug transporters, such as P-gp and BCRP [32,41].

Asciminib is the first STAMP (specifically targeting the ABL1 myristoyl pocket) inhibitor that has granted breakthrough therapy designation by FDA in 2021. This approval was based on ASCEMBL trial results and intend to adult Ph-positive CML in CP previously treated with two or more TKIs or patients harboring the T315I mutation. In opposition to the previously described TKI, Asciminib targets the myristoyl site of ABL1 kinase with an in vitro IC_50_ of 1–20 nM and from 40 to 200 mg twice a day in trials [46,243,244]. By targeting different portions of ABL1 kinase, Asciminib may be very useful to overcome TKI resistance mediated by point mutations previously mentioned (Section 2.1). However, for this TKI were already identified mechanisms that could lead to resistance, namely point mutation at myristylation pocket (V468F and I502L) and the function of ABC transporters [245,246]. Due to different mechanisms of action on the same kinase, the combination of the other approved TKI with Asciminib has been explored with the aim to reduce the possible appearance of BCR-ABL1 mutant clones [37,247].

New TKIs have been designed to overcome ABL1 gatekeeper mutations (mainly T315I) and at present are only preclinically validated inhibitors, such as Bafetinib, Rebastinib, Tozasertib, and Danusertib [248]. Other new molecules are already under clinical trials e.g., PF-114, HQP1351 (Olverembatinib), and K0706 (Vodobatinib), which function competitively as inhibitors of BCR-ABL1 TK at the ATP-binding site (Table 2) [249]. These inhibitors have presented an increased potency against a wide range of BCR-ABL1 mutations [249,250,251,252] and may overcome some the limitations of approved TKIs. Unlike other TKIs, Olverembatinib is able to bind to the kinase in the presence of T315I mutations since it does not form the hydrogen bond with the hydroxyl group at this residue [251]. On the contrary, PF-114 is structurally very similar to Ponatinib but modified to avoid the VEGFR inhibition associated with cardiovascular side effects [252].

### 4.2. Non-BCR-ABL1 Targeted Therapies

Despite the considerable success of BCR-ABL1 inhibitors in CML, even with second and third-generation TKI in the clinical setting, this therapeutic protocol is not a curative approach. The increased knowledge of CML biology, especially with recognition of dormant LSCs, highlights the necessity to explore non-BCR-ABL1 targets [199,253]. Over the years, multiple new agents, and “old” drugs with a new purpose (drug-repurposing) have been studied in monotherapy or in association with TKIs, to promote a synergistic effect, trying to induce CML cell death.

As mentioned before, a critical protein in the transduction of BCR-ABL1 oncogenic signal is GRB2, which is able to activate RAS/MAPK, JAK/STAT, and other signaling pathways resulting in cell proliferation stimuli [254]. BP1001 is a liposome incorporated GRB2 antisense oligonucleotide developed to inhibit GRB2 expression. This agent was under phase I clinical trial in CML and other hematological cancers (NCT01159028), and in phase II study in combination with Dasatinib (NCT02923986) [255]. Despite the promising results in preclinical studies and being well tolerated in patients, the BP1001 effect in combination with Dasatinib was insufficient, and the phase II trial was withdrawn. However, this approach is currently under investigation in AML and solid tumors [256].

The RAS/MEK/ERK pathway can be activated independently of fusion oncoprotein in response to growth factors [257]. An initial step in RAS activation is the transfer of a farnesyl group mediated by farnesyltransferase [258]. Tipifarnib (NCT00004009) and Lonafarnib (NCT00047502), two farnesyltransferase inhibitors, were tested in CML resistant patients in monotherapy and in combination with Imatinib [259,260,261]. Although some positive results have been observed, the clinical interest in these inhibitors for CML treatment was ceased. Currently, Lonafarnib is approved for progeria and other conditions [262]. Other agents that modulate the RAS/MEK pathway were also explored as potential CML therapeutic options. These drugs include Selumetinib (NCT03326310) and Trametinib, both MEK inhibitors, and Enzastaurin—a PKC inhibitor [263,264].

Ruxolitinib, a JAK2 inhibitor currently approved for myelofibrosis and polycythemia vera, demonstrated promising results in combination with TKI in reducing LSC viability in CML [265]. Its mechanism of action is associated with direct inhibition of JAK signaling but is also linked with enhancing MHC molecules expression making CML cells more visible to the immune system [210]. The combination approach of Ruxolitinib with TKI is currently under investigation in different clinical trials (NCT03610971, NCT01702064).

The recognition of the PI3K/AKT/mTOR pathway as an important drug target in oncology led to the approval of PI3K inhibitors (as Idelalisib) and mTOR inhibitors (as an example of Sirolimus) for different neoplasias [266]. In preclinical studies, Everolimus, an mTOR inhibitor, show promising results overcoming TKI resistance in cell lines and ex vivo samples [267,268]. Based on these results, Sirolimus (NCT00776373) and Everolimus (NCT00081874 and NCT01188889) were evaluated in phase I and II clinical trials in CML patients [269]. However, the trials were terminated and completed, respectively, without published results and an evaluation of the obtained outcomes. Other agents targeting this signaling axis have been explored in preclinical studies, namely Pictilisib (PI3K inhibitor) and MK-2206 (AKT inhibitor) [124,270].

BCL-2 family members became druggable targets in hematopoietic cancers to overcome the anti-apoptotic signal in tumor cells [271]. Venetoclax, a specific BCL-2 inhibitor, demonstrated in preclinical tests an increase in the apoptotic rate in CML cells and presented a synergistic effect with TKI against CD34-positive CML cells [272,273]. Approved for CLL treatment, Venetoclax is currently in a phase II trial in resistant CML patients in combination with Dasatinib (NCT02689440) and in another two trials using a triple combination with Ponatinib and Decitabine (NCT04188405) or Ponatinib and corticosteroids (NCT03576547).

In cancer cells, the repression of tumor suppressor proteins, like P53, leads to uncontrolled proliferation, resistance to apoptosis, and survival. The degradation of P53 is mediated by MDM2, an E3 ligase that targets the protein to the proteasome. Inhibition of MDM2 may constitute a critical approach to restore P53 function and control tumor cell fate indirectly [274]. Different MDM2 inhibitors showed satisfactory effects in vitro, and AMG-232 (KRT-232) is currently in clinical trials associated with Dasatinib and Nilotinib in CML patients (NCT04835584) [275]. Another way to overcome TKI resistance is by the inhibition of protein translation [276]. In 2012, the FDA approved the use of Omacetaxine, a protein translation inhibitor, to treat resistant CML patients that do not benefit from TKI therapy [277]. By inhibiting the synthesis of oncoproteins as BCR-ABL1, Omacetaxine presented anti-tumor activity against CML cells and showed meaningful response rates in clinical trials, even in the case of T315I mutation [278,279].

Targeting signaling pathways essential to maintain and regulate key features of LSCs is a very promising strategy in different cancers [210]. Inhibition of sonic hedgehog pathway with Vismodegib (GDC-0449) or Sonidigib (LDE225) in preclinical studies showed a reduction in number and self-renewal capacity of CML-LSCs and a possible synergistic effect with TKI [280,281]. Although the trial results are not posted yet, Sonidigib was investigated in combination with Nilotinib in CML patients resistant to prior treatments (NCT01456676). Additionally associated with quiescent LSC state and its insensitivity to TKI, Pioglitazone may gain a new therapeutic purpose [282]. Used as an antidiabetic drug in type 2 diabetes patients, Pioglitazone is a peroxisome proliferator-activated receptor gamma (PPAR-γ) agonist. Several clinical trials are testing this drug in combination with TKIs (NCT02889003, NCT02687425) or other drugs (NCT02767063). The rationale behind this combination with TKI is based on the capability of Pioglitazone to induce CML LSC to exist their quiescent state and become sensitive to TKI therapy [282]. Mechanistically, this PPAR-γ agonist downregulates STAT5 and consequently HIF2α and CITED2, crucial regulators of the quiescence and stemness state of CML LSC cells [283]. In combination with TKI, Pioglitazone is in clinical trials to evaluate its importance not only in resistance but also in improving TKI discontinuation and TFR rates.

Many other molecules and pathway inhibitors were or are currently in preclinical studies trying to overcome resistance and may constitute future therapeutic options in CML. The exploitation of other signaling pathways either alone or in combination with BCR-ABL1 drugs will, sooner or later, become a reality in CML treatment to improve response, avoid resistance, and enhance treatment discontinuation probability.

### 4.3. Epigenetic Modulators

The fact that epigenetic modifications can be manipulated pharmacologically has led to their successful use in clinical practice in both myeloid and lymphoid malignancies.

The two most commonly used DNA hypomethylating agents are Azacitidine and Decitabine and both have been particularly successful in the treatment of MDS [284,285,286] and AML [287,288]. Decitabine has been tested as both first and second line in CML patients. One hundred and thirty naïve CML patients were treated with escalating doses of Decitabine, having achieved hematological responses in a significant number but at the cost of prolonged myelosuppression [289]. Lower doses of Decitabine were tested in a phase 1 trial of treatment naïve CML patients with better hematological and cytogenetic responses [290]. In Imatinib-resistant CML patients, Decitabine was tested in combination with higher doses of Imatinib, achieving hematologic responses in 30–50% patients [291]. Unfortunately, none of these combinations demonstrated sufficient efficacy to justify their clinical use.

Histone modifications can also be manipulated pharmacologically, especially with HDAC inhibitors. These include aliphatic acids (phenylbutyrate), cyclic peptides (romidepsin), benzamides (entinostat), electrophilic ketones, and hydroxamates (vorinostat) and may restore normal acetylation of histone proteins and transcription factors [292]. Some HDAC inhibitors have been successful in treating hematological malignancies, such as vorinostat for cutaneous T-cell lymphomas [293]. There is also in vitro evidence that vorinostat has a significant effect in chronic myeloid malignancies [294] and may act synergistically with TKI to induce p21 and p27 expression and inhibit BCR-ABL1 levels [295,296]. However, there are no firm clinical data to corroborate these laboratory findings, for the use of these agents in CML.

### 4.4. Immunotherapies

During the past decade, considerable progress has been made in the immunology understanding of CML, raising hopes that this disease may be curable by improving the currently targeted chemotherapy with immunotherapeutic approaches [297]. Immune responses against CML-specific and CML-associated antigens such as BCR-ABL1, proteinase-3, and WT-1 can be detected in CML patients, suggesting it sensitivity to immune control. Besides that, donor lymphocyte infusions can induce long-lasting remissions in relapsed CML patients after allogeneic stem cell transplant (SCT) [247]. Through the use of T cell-based immunotherapy, CML-specific immune responses may be strengthened, extending the fraction of patients achieving long-term TFR or even complete cure. It has been shown that “non-specific” immunotherapy approaches, such as allogeneic stem cell transplantation or interferon-α (IFN-α) therapy, enable long-lasting remissions in CML patients after discontinuation of TKI therapy [298]. The anti-leukemic effect of IFN-α is through a direct anti-proliferative effect, specifically on CML progenitor cells, but it also has an immunomodulating action. In clinical practice, in the TKI era, interferon in monotherapy has a limited place and is mainly used for patients who were TKI intolerant or that could not be treated with a TKI (for example during pregnancy) [247]. With the advent of pegylated formulations, having higher tolerability, interferon has re-emerged as an attractive therapeutic option in CML [2]. Besides that, and because of their different modes of action, exploration of the potential to combine interferon with TKI therapy is also a possibility, being the pegylated form of interferon in combination with the second-generation TKIs, Nilotinib and Dasatinib (NCT01866553, NCT01872442), and with Bosutinib (NCT03831776) [212,247,249].

Several cooperative groups and trials showed significantly higher complete cytogenetic remissions and major molecular remission rates for patients treated with Imatinib in combination with IFN-α as compared with patients treated with Imatinib alone (Italian GIMEMA, Nordic CML study group French SPIRIT trial, German CML IV trial). However, the interferon discontinuation rate in all studies was high (83%), mainly due to toxicity. None of those studies mentioned above found a statistically significant reduction in progression to advanced disease phase or prevention of CML-related death [247].

In CML patients, particularly in those classified as high risk by the Sokal score, the expression of the immune checkpoint proteins PD-L1 and PD-1 had been observed. Thus, the upregulation of PD-L1 is considered an immunological escape mechanism for CML cells [247,249]. These data suggest that targeting the PD-1/PD-L1 pathway may be an effective strategy for eliminating CML cells [249], and treatment with ICIs could potentially increase immunoreactivity against leukemic cells in CML [4]. Several clinical trials have evaluated the combination of ICIs with TKI therapies (NCT02011945, NCT02767063, NCT03516279, NCT01822509) [204]. Monoclonal antibodies, such as Pembrolizumab, may interfere with the ability of cancer cells to grow and spread. Administrating Pembrolizumab in combination with TKIs may be more effective in treating patients with CML. A clinical trial (NCT01822509) is currently evaluating the efficacy of Ipilimumab (anti-CTLA-4) in combination with Nivolumab (anti-PD1) in patients with hematological malignancies, including CML [204]. Although the mechanism of action of the ICIs in CML is unclear, it has been demonstrated, in murine models, that the therapeutic effects of PD1/PDL1 blockade may be mediated, at least in part, through a strong NK response [204].

More advanced therapeutic strategies, e.g., vaccines or engineered T cells, are in study to treat CML patients aiming to induce an immune response against the leukemic cells. In general, tumor-associated HLA-presented peptides on malignant cells are relevant targets. However, the role of these neoantigens in cancers with a low mutational burden, such as CML, remains unclear. Several small studies have investigated the potential to induce an anti-leukemic vaccination response in CML patients. One strategy uses ex-vivo generated autologous dendritic cells and other leukemia-associated antigens as the injection of BCR-ABL1 derived peptides (NCT02543749). This strategy has shown clinical efficacy by inducing a T-cell response in most patients and was safe and feasible. However, these studies are single arm and, therefore, prospective randomized trials are needed [247].

Chimeric antigen receptor (CAR)-T cells are an emerging immunotherapy already approved for B-cell malignancies and are being evaluated for myeloid malignancies. The IL1 receptor accessory protein (IL1RAP), a co-receptor for the IL1 and IL33 receptors, is a cell-surface marker expressed by CML cells but not by normal HSCs. In vitro and in vivo studies suggest that CART-cells targeting IL1RAP specifically induce cell death of quiescent CML stem cells and have a favorable side effect profile without off-target toxicity or tumor lysis syndrome, some of the adverse events more commonly associated with CAR-T therapy. A combined CAR-T and TKI approach has also been used to eliminate CML stem cells in limited clinical series. An anti-CD19 CAR-T therapy combined with Dasatinib induced complete molecular remission and the return to CP in CML patients in lymphoid BC harboring T315I mutation. This CART cleared the T315I mutation and re-sensitized cells to Dasatinib. The same effect was observed against *RUNX1* mutations in BC-CML patient, where the anti-CD19 CAR-T showed an additive effect when combined with Imatinib. These studies suggest that CART-cells may confer therapeutic benefit, particularly in young or advanced-phase CML patients who are resistant/intolerant to TKI, highlighting the critical importance of the immune system in optimizing treatment responses in CML [204].

### 4.5. Allogenic Stem Cell Transplantation

In TKI pre-era allogenic stem cell transplantation (Allo-SCT) represented a therapeutic option for patients at CP-CML with a compatible donor and fit for the procedure [299]. However, currently this option has a more important role for patients that evolve into AP/BP-CML and remains an important therapeutic option for CP-CML patients that presented resistance to second line TKI or first line TKI resistance with T315I mutations, in agreement with ELN guidelines [212]. According to expert opinions, in case of second line TKI resistance the search for a compatible donor should be initiated as early as possible. This is justified by the time taken until find an unrelated donor, since two thirds of patients do not have a matched-related donor [300].

The appropriated time for perform Allo-SCT has not established yet, and the decision must be based on patient’s individual benefit–risk assessment. The multiple transplant-associated risks, such as non-relapse mortality and graft versus host disease, and the presence of high-risk ACA are some of the factors to be considered in patient evaluation [299]. In case of young patients, the transplant should be preferred to a third line TKI, such as Ponatinib, if a donor is available [300]. This therapeutic approach is an effective alternative for TKI-resistant or intolerant patients. However the strategies to adopt after Allo-SCT to avoid relapse continue to be poorly elucidated [301,302].

## 5. Patient’s Adhesion Impact on Resistant Process

In CML treatment, the introduction of TKIs had been considered a revolutionary and successful therapeutic, leading to a normal life expectancy. However, a substantial proportion of CML patients fail treatment with drug interruption and discontinuation, rapidly leading to disease resurgence due to minimal residual disease re-emergence [303] and probably contributing to drug resistance development.

In this context, adherence to therapy and compliance with clinical instructions are critical in the management of CML. Adherence to long-term therapy is defined by the World Health Organization (WHO) as “the extent to which patients’ behavior taking medication corresponds with agreed recommendations from a healthcare provider” [304]. The adherence rates to oral anticancer therapies vary greatly, ranging between 0% and 83%, with an average non-adherence rate estimated at 21%. These variations can be partly explained by differences in measurements and definitions of non-adherence [16]. There are diverse methods previously described to verify adherence being the most common the medication possession ratio (MPR), the continuous measure of adherence (CMA), and the proportion of days covered (PDC) [17].

Several studies have shown that lack of adherence to Imatinib is frequent and may significantly impact patient outcomes. A Belgian study found that only 14% of CML patients took all of their prescribed Imatinib. This lack of compliance led to increased suboptimal responses [305]. Others show that 26% of the patients on long-term Imatinib therapy have an adherence rate lower than 90% being this the most important factor determining the achievement of molecular responses [306]. In addition, a study in the US found that 31% of 267 CML patients were identified as having treatment interruptions of Imatinib at least 30 consecutive days during a year follow-up period and that this non-adherence increased health care costs [307]. A study performed in Qatar showed a high rate of treatment failure explained by poor adherence, economic factors being the main causes of non-adherence [303]. In vitro models exposed to discontinuous TKI, which mimics the consequence of poor adherence or other causes of treatment interruptions, emphasizes the complexity of the Imatinib resistance process [87]. As described, the low adherence to Imatinib is a common problem in clinical practice being a significant risk of therapeutic failure and Imatinib resistance as well as for increased health care services costs [17].

The factors that seem to facilitate adherence are fitting the Imatinib into the daily routine, using prompts to remember to take the tablets and finding ways of coping with side effects. Another intervention that may help reduce intentional non-adherence is making a patient phone call and discussing how they are getting on with their medication. The interventions to improve adherence need to consider both intentional and unintentional reasons for not taking Imatinib and should target the specific personal reasons for not correctly receiving TKI treatment [16].

## 6. Conclusions

Chronic myeloid leukemia was a pioneer in terms of targeted treatment approaches [308]. The development of tyrosine kinase inhibitors able to counteract the function of BCR-ABL1 oncoprotein improved the survival of CML patients substantially [308,309], changing the natural course of this disease [309,310]. Despite the spectacular progress made over the last two decades to obtain new TKIs that target CML, multiple patients still develop resistance to these drugs.

The molecular mechanisms behind TKI resistance are multiple, ranging from changes in the molecular drug target itself, to mechanisms that alter drug concentration or modify leukemic cells signaling network [23]. In addition, the modifications on tumor microenvironment and immune cell dysfunction may compromise drug response(s). Alterations in drug target are the most common mechanism of resistance in CML, but are not exclusive. Exploiting *BCR-ABL1* independent mechanisms seems to be important to understand and identify the role of other proteins in treatment response [27]. Identification of biomarkers of drug response are crucial for a better treatment selection and some of them may constitute new targets for future therapeutic approaches [180]. Additionally, inherent genetic variants, such as SNVs, may modulate or affect the predisposition to disease, prognosis, and drug response [311].

In this context, blood stem cell transplant therapy is the only proven cure for these patients, but this therapy has higher toxicity and is limited by donor availability [212,312]. This fact highlights the need for the development of new therapeutic approaches against resistance for CML treatment. The point mutations in BCR-ABL1 chimeric protein, including the gatekeeper T315I mutation, are the principal cause for the development of resistance to TKIs. However, other mechanisms are also involved in the failure of TKI therapy [312]. Considering these causes of therapy failure, several strategies have been used to overcome drug resistance in CML: (I) the use of drugs targeting the allosteric site of BCR-ABL1 oncoprotein; (II) the use of drugs targeting the ATP site of BCR-ABL1 along with drugs that bind in a different way to Imatinib; (III) the use of drugs, like Asciminib, that target the myristoyl pocket of BCR-ABL1; (IV) the combined use of several TKIs; (V) the use of a TKI in combination with other drugs that target different objectives, such as TKI+IFN-α, TKI+chemotherapy, TKI+immune-modulators, etc. [210]; (vi) the use of new TKIs designed to overcome ABL1 gatekeeper mutations (mainly T315I) that at present are only preclinically validated [248]. All these therapeutic approaches are taken in second and third line treatments when initial therapies are not efficient or faint over time due to the emergence of resistance.

The emerging landscape of immune dysfunction and immunosurveillance in CML highlights the critical importance of the immune system in optimizing treatment responses in CML. Curiously, IFN-α, a previous standard of care therapy, exhibits non-specific, untargeted effects on the immune system, leading to repurpose it in order to enhance TFR in the TKI era. However, newer precision oncology approaches targeting immune system, such as vaccines, ICIs, and CAR-T-cell therapies constitute a great promise in CML therapy, especially in cases of TKI resistance and/or intolerance. Together, specific and non-specific immunological effectors can make the concept of "operational cure" a reality for the vast majority of patients in the next decade [204].

## Figures and Tables

**Table 1:** Most frequent *BCR-ABL1* mutation and the sensitivity degree to the approved TKIs.

*BCR-ABL1* Mutation	Location ^$^	Imatinib	Dasatinib	Nilotinib	Bosutinib	Ponatinib	Asciminib
Wild-type		Sen	Sen	Sen	Sen	Sen	Sen
M244	P-loop	Sen	Sen	Sen	Sen	Sen	Sen
L248	P-loop	Int	Int	Sen	Int	Sen	Sen
G250	P-loop	Res	Sen	Int	Res	Sen	Sen
Q252	P-loop	Int	Int	Sen	Sen	Sen	Sen
Y253 ^§^	P-loop	Res	Sen	Res	Sen	Sen	Sen
E255 ^§^	P-loop	Res	Int	Res	Int	Int	Sen
V299	C-helix	Res	Res	Sen	Res	Sen	Sen
T315 ^‡, §^	Drug contact site	Res	Res	Res	Res	Int	Sen
F317 ^‡^	Drug contact site	Int	Res	Sen	Int	Sen	Sen
A337	C-loop	Sen	Sen	Sen	Sen	Sen	Int
M351	C-loop	Sen	Sen	Sen	Sen	Sen	Sen
M355	C-loop	Int	Sen	Sen	Sen	Sen	Sen
F359 ^§^	C-loop	Int	Sen	Res	Sen	Sen	Sen
H396	A-loop	Int	Sen	Int	Sen	Int	Sen
W464	Myristate pocket	Sen	Sen	Sen	Sen	Sen	Res
P465	Myristate pocket	Sen	Sen	Sen	Sen	Sen	Res
V468	Myristate pocket	Sen	Sen	Sen	Sen	Sen	Res
I502	Myristate pocket	Sen	Sen	Sen	Sen	Sen	Res

**^‡^** Gatekeeper residue; **^§^** Most commonly associated with disease progression and relapse; **^$^** Location of altered amino acid on BCR-ABL protein, P-loop: ATP-binding loop; C-loop: catalytic loop; A-loop: activation loop; Sen: sensitive; Int: intermediate sensitivity; Res: resistant. Data derived from the following references: [27,32,34,35].

**Table 2:** Selected clinical trials of different therapeutic strategies in CML.

Drug	Class/Mechanism of Action	NCT Number	Scheme	Phase
**BCR-ABL1 therapies**				
Asciminib	ABL1 myristoyl pocket inhibitor	NCT02081378	Mono. and Comb. TKI	1
		NCT03595917	Plus Dasatinib	1
		NCT03906292	Mono. and Comb. TKI	2
		NCT03578367	Plus Imatinib	2
		NCT03106779	Mono.	3
		NCT04971226	Mono.	3
		NCT04948333	Mono.	3
		NCT04877522	Mono. and Comb. TKI	3
		NCT04795427	Mono.	2
		NCT04666259	Mono.	3
Flumatinib	BCR-ABL1 ATP-binding site inhibitor	NCT04677439	Mono.	4
		NCT04933526	Mono.	4
PF-114	BCR-ABL1 ATP-binding site inhibitor	NCT02885766	Mono.	1/2
HQP1351	BCR-ABL1 ATP-binding site inhibitor	NCT03883100	Mono.	2
		NCT03883087	Mono.	2
		NCT04126681	Mono.	2
		NCT04260022	Mono.	1
Vodobatinib	BCR-ABL1 ATP-binding site inhibitor	NCT02629692	Mono.	1/2
**Non BCR-ABL1 therapies**				
BP1001	GRB2 antisense oligonucleotide	NCT01159028	Mono.	1
		NCT02923986	Plus Dasatinib	2
Tipifarnib	Farnesyltransferase inhibitor	NCT00004009	Mono.	1
Lonafarnib	Farnesyltransferase inhibitor	NCT00047502	Plus Imatinib	1
Selumetinib	MEK inhibitors	NCT03326310	Plus Azacitidine	1
Ruxolitinib	JAK2 inhibitor	NCT03610971	Comb. TKI	2
		NCT01702064	Plus Nilotinib	1
Everolimus	mTOR inhibitor	NCT00081874	Mono.	1/2
		NCT01188889	Plus Imatinib	2
Sirolimus	mTOR inhibitor	NCT00776373	Plus Citarabine	1/2
Venetoclax	BCL-2 inhibitor	NCT02689440	Plus Dasatinib	2
		NCT04188405	Plus Ponatinib, Decitabine	2
		NCT03576547	Plus Ponatinib, corticosteroids	1/2
AMG-232	MDM2 inhibitor	NCT04835584	Mono.	1/2
Sonidigib	SHH inhibitor	NCT01456676	Plus Nilotinib	1
Pioglitazone	PPAR-γ inhibitor	NCT02852486	Plus Imatinib	2
		NCT02767063	Plus TKI	1/2
		NCT02889003	Plus TKI	2
**Epigenetic modulators**				
Azacitidine	Hypomethylating agent	NCT03895671	Plus Ponatinib	2
		NCT01460498	Plus TKI	1
Panobinostat	Histone Deacetylase Inhibitor	NCT00451035	Mono.	1
**Immunotherapies**				
Peg-IFNα	Pegylated Interferon alpha	NCT01866553	Plus Nilotinib	2
		NCT01872442	Plus Dasatinib	2
		NCT03831776	Plus Bosutinib	2
Nivolumab	Anti-PD-1 antibody	NCT02011945	Plus Dasatinib	1
		NCT01822509	Plus Ipilimumab	1
Pembrolizumab	Anti-PD-1 antibody	NCT03516279	Plus TKI	2
DC vaccine	Dendritic cells vaccine	NCT02543749	Mono.	1/2
CLL1-CD33 cCART	compound CAR (cCAR) T cells	NCT03795779	Mono.	1
KDS-1001	natural killer cell therapy	NCT04808115	Plus TKI	1

Comb: combination; Mono: monotherapy; NCT: national clinical trial number; TKI: tyrosine kinase inhibitors.
